# Synthesis and anti-melanoma effect of 3-O-prenyl glycyrrhetinic acid against B16F10 cells *via* induction of endoplasmic reticulum stress-mediated autophagy through ERK/AKT signaling pathway

**DOI:** 10.3389/fonc.2022.890299

**Published:** 2022-08-02

**Authors:** Lone A. Nazir, Naikoo H. Shahid, Kumar Amit, Sheikh A. Umar, Sharma Rajni, Sandip Bharate, Pyare L. Sangwan, Sheikh Abdullah Tasduq

**Affiliations:** ^1^ Pharmacokinetics-Pharmacodynamics and Toxicology Division, Council Of scientific and Industrial Research-Indian Institute of Integrative Medicine, Jammu, India; ^2^ Academy of Scientific and Innovative Research (AcSIR), Ghaziabad, India; ^3^ Natural Product and Medicinal Chemistry Division, CSIR-Indian Institute of Integrative Medicine, Jammu Tawi, India

**Keywords:** melanoma, ER stress, autophagy, apoptosis, 3-O-prenyl glycyrrhetinic acid

## Abstract

Melanoma is an aggressive form of cancer with poor prognosis and survival rates and limited therapeutic options. Here, we report the anti-melanoma effect of 3-O-prenyl glycyrrhetinic acid (NPC-402), a derivative of glycyrrhtinic acid, from a reputed medicinal plant *Glycyrrhiza glabra* against B16F10 cells. We studied the cytotoxic effect of NPC-402 on melanoma cells and investigated the role of mitogen-activated protein (MAP) kinase, AKT axis, and endoplasmic reticulum (ER) stress/unfolded protein response (UPR)-mediated autophagy as the involved signaling cascade by studying specific marker proteins. In this study, 4-phenylbutyric acid (4PBA, a chemical chaperone) and small interference RNA (siRNA) knockdown of C/EBP Homologous Protein (CHOP)/growth arrest- and DNA damage-inducible gene 153(GAD153) blocked NPC-402-mediated autophagy induction, thus confirming the role of ER stress and autophagy in melanoma cell death. NPC-402 induced oxidative stress and apoptosis in melanoma cells, which were effectively mitigated by treatment with N-acetylcysteine (NAC). *In vivo* studies showed that intraperitoneal (i.p.) injection of NPC-402 at 10 mg/kg (5 days in 1 week) significantly retarded angiogenesis in the Matrigel plug assay and reduced the tumor size and tumor weight without causing any significant toxic manifestation in C57BL/6J mice. We conclude that NPC-402 has a high potential to be developed as a chemotherapeutic drug against melanoma.

## Graphical Abstract

Graphical summary of NCP-402 effect on endoplamis reticulum and autophagy. NPC-102 induces ER, stress lading to UPR via elF 2a signaling, resulting in activabos of CHIOP and Causes aulophagy induction mediated cell death. NPC-402 causes generation of reactive axygen species that leads to MMP depolarization resulting in pro-apoptolic environment within the cells, NPC-d02 alo cause's felease of cylochrone C nio eytosol that in turn actrales irininsic apoptotic puthway. Application of chemical chaperone 4 Phenyl butyric acid (APBA) interferes will NPC-402 -induced ER-ares and its downward effects, N acetyl eysteine alio preveat NCP,A03 indaced cell death 5) relieving oxidative stres.

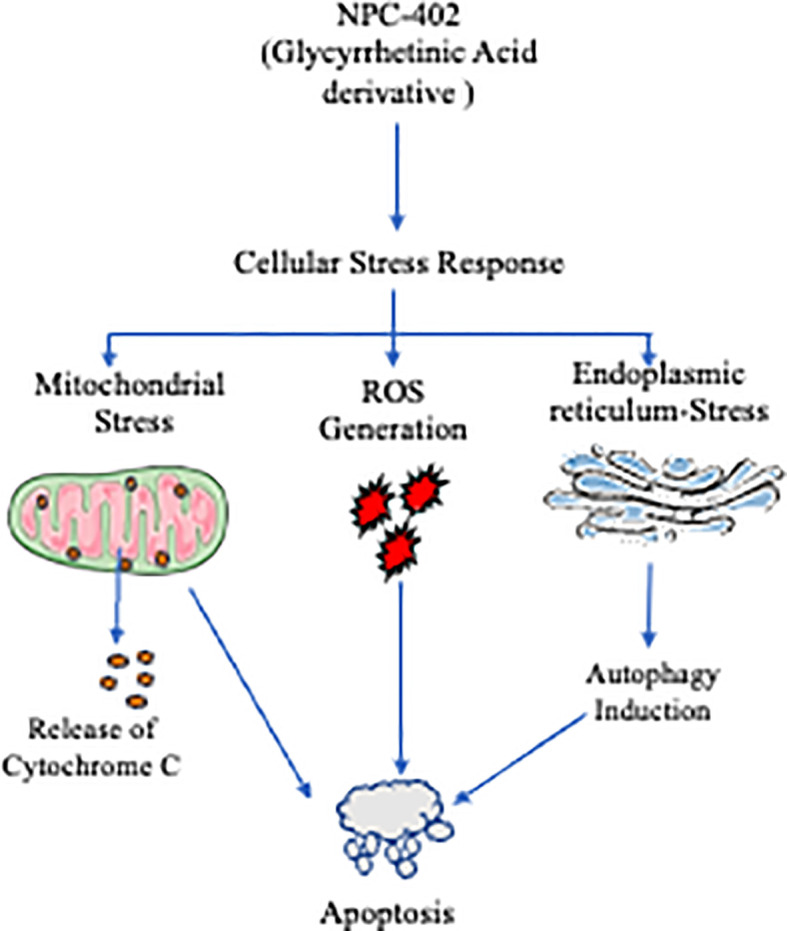

## Introduction

Melanoma is the deadliest cancer among all skin cancers, with high metastasis, poor prognosis, and resistance to present treatment strategies (1). It represents less than 5% of skin malignancies but accounts for 80% of related mortalities (2). Due to its extraordinary opposition to conventional chemotherapeutic strategies, there is urgency for the development of novel and targetable therapeutic strategies to control this deadly disease (3). A high load of oxidative stress in melanoma tumors increases the aggressiveness of therapeutic agents imparting a high Reactive Oxygen Species(ROS) stress threshold in cancer cells and has shown promising potential to selectively target melanoma (4, 5). phosphoinositide 3-kinase(PI3K)-AKT has been shown to increase in part the proliferative and anchorage-independent growth nature in melanoma cells (6, 7). Moreover, mitogen-activated protein kinase (MAPK) signaling has been found to be critical and constitutively activated by various mechanisms, making it a target for melanoma (8).

Endoplasmic reticulum (ER) is a membrane-bound cytosolic organelle with a high calcium (Ca^2+^) storage and neutral pH and plays a pivotal role in protein folding due to the presence of chaperone proteins such as glucose-related protein (GRP)78/Bip collectively controlling cellular homeostasis. A hostile cellular environment causes misfolded proteins to accumulate, leading to imbalances in ER homeostasis and finally culminates into ER stress/unfolded protein response (UPR). The signaling pathways overseeing the ER stress response are inositol-requiring transmembrane kinase/endoribonuclease 1α (IRE1α), protein kinase R-like endoplasmic reticulum kinase (PERK), and activating transcription factor (ATF)6. UPR results in the induction of the expression of proteins called chaperones that are involved in the recovery process. However, in case of excessive stress or sustained insult, the function of the ER is compromised, triggering apoptosis, thereby removing the affected cell. Recent findings in this regard have well established that ER stress is a potent inducer of autophagy, a cellular self-degradative process having an adaptive function (9), and modulation of UPR by agents that actively induce ER stress may be effective as anti-melanoma agents (10, 11). These pieces of evidence are highly suggestive of ER stress-mediated autophagy as a therapeutic target against melanoma.

During physiological conditions, autophagy plays a housekeeping role in removing the misfolded proteins and in clearing damaged organelles from cells (12, 13). Previous studies have suggested that autophagy performs context-dependent roles in cell survival and death mechanisms (14). In cancer morbidity conditions, autophagy deficiency acts as a tumor suppressor or supports tumor progression by limiting stress (15). Induction of autophagy on stimulation can either be protective or promote apoptosis in cancer cells. Therefore, anticancer drugs acting through autophagy in tumors leads to autophagy-mediated cell death (16), and strategies targeting autophagy as a drug target are worth it in melanoma cancer therapy. In melanoma, c-Jun N-terminal kinase (JNK) signaling pathway has a major role to play in autophagy-mediated apoptotic cell death (17, 18). Therefore, it has been clearly inferred that autophagy and MAPK/JNK along with AKT signaling axis can also be targeted to induce autophagy-mediated cell death response in melanoma cells (19).

Nature-based agents have been the backbone of drug discovery in the area of cancer treatment strategies. Different derivatives of 18β-glycyrrhetinic acid, a secondary metabolite from a reputed plant used in most traditional medicine practices of the world, *Glycyrrhiza glabra*, are in various phases of clinical trials against various cancers (20, 21). In the present study, we synthesized and analyzed the anti-melanoma effect and the mechanistic basis of 3-(3-methyl-but-2-enyloxy)-11-oxo-olean-12-ene-29-oic acid (NPC-402), a pentacyclic triterpenoid ether derivative of glycyrrhetinic acid (GA), against several cellular models of melanoma and in C57BL/6J mice. The present study was designed to demystify the mechanistic basis and interrelationship of ER stress and autophagy in cell death by examining and looking for the changes in the PERK–ATF4–C/EBP Homologous Protein (CHOP) and MAPK and PI3K/AKT signaling pathway axes regulating cellular proliferation and death processes and evaluating their role as therapeutic targets for NPC-402 treatment to achieve an anti-melanoma effect. In addition, a reduction in tumor size/volume in C57BL/6J mice in the Matrigel plug assay was performed to assess the efficacy and potency of our study molecule (NPC-402) as an anti-melanoma agent.

## Materials and methods

### Chemicals and reagents

All of the chemicals/reagents were purchased from different authorized companies. Most of the cell culture-based chemicals and reagents were purchased from Sigma-Aldrich, and the cultureware was purchased from the Nunclon Thermo-Scientific-India. The Western blot apparatus, tools, and reagents were purchased from Bio-Rad Technologies, USA. Dulbecco’s modified Eagle’s medium (DMEM), penicillin–streptomycin, propidium iodide (PI), 2,7-H_2_DCF-DA, proteinase K, ribonuclease-A, agarose, dimethyl sulfoxide (DMSO), Dulbecco’s phosphate buffer saline (DPBS), fetal bovine serum (FBS), LC3I/II, and anti-β-actin antibodies were purchased from Sigma-Aldrich Chemicals (St. Louis, MO, USA). Antibodies against caspase-3/caspase-8, B-cell lymphoma 2 (Bcl-2), Bcl-2 homology 3-only protein (Bim), BCL2-associated X (Bax), ATF4, phospho-mitogen-activated protein kinase kinase (MEK), phospho-extracellular signal-regulated kinase (ERK), total ERK, total MEK, phospho-p38, p-38, phospho-JNK, JNK, phospho-AKT, nuclear factor erythroid 2-related factor 2 (Nrf-2), catalase, copper/zinc superoxide dismutase (Cu/Zn SOD), AKT, Beclin1, P62, and glyceraldehyde 3-phosphate dehydrogenase (GAPDH) were purchased from Santa Cruz Biotechnology, Inc. Glycyrrhizin was purchased from Sigma-Aldrich.

### Synthesis and characterization of 3-(3-Methyl-But-2-Enyloxy)-11-Oxo-Olean-12-Ene-29-Oic Acid (NPC-402)

#### Hydrolysis of glycyrrhizin

GA was prepared by hydrolyzing glycyrrhizin in 5% aq. HCl and purified over a silica column in ethanol:hexane (25:75) and further characterized by comparison of melting point and spectral data with values in literature. The modifications were done at the C-3 position for the synthesis of *O*-alkylated/benzylated GA derivatives.

#### General method for the preparation of *O*-Alkylated/Benzylated Glycyrrhetinic acid

K_2_CO_3_ (1.2 mmol) was added to the solution of 3β-hydroxy-11-oxo-olean-12-en-30-olic acid (100 mg, 1 mmol) in dry acetone (5 ml) followed by addition of different alkyl and benzyl halides (1 mmol). Stirring was done at room temperature for 8 h under inert atmosphere and concentrated under reduced pressure conditions. The resultant mixture was chloroform diluted and water was added, leading to the formation of a white precipitate in the aqueous layer. The organic layer was decanted, and the remaining residual solid material was washed 5–6 times with chloroform. Under reduced pressure, the combined chloroform layer was evaporated and the residue was purified by silica gel (#100-200) column chromatography using hexane-ethyl acetate as an eluent to yield the different alkylated products (**Figures S1A, B**).

### Cell culture and animal experiments

Murine melanoma cell line B16F10, human immortalized fibroblast cell line from foreskin Hs68, and cell line A375 (human melanoma) were purchased from the American Type Culture Collection (Rockville, MD, USA). Human immortalized keratinocyte cell line HaCaT was purchased from CSL-Eppelheim Germany. The cell culture method was followed as described previously (22). Briefly, cells were seeded in DMEM supplemented with L-glutamine, glucose (3.5 g/L), and (4-(2-hydroxyethyl)-1-piperazineethanesulfonic acid) (HEPES) (15 mM). Additionally, penicillin G (120 mg/L), streptomycin (270 mg/L), amphotericin B (250 mg/L), sodium bicarbonate 1.2 g/L, sodium pyruvate 220 mg/L, and FBS (10% v/v) were added. The culture conditions for all cell culture experiments were 37°C and 5% CO_2_ and kept for incubation in a water jacketed humidified incubator (Thermo-Scientific Model No. 3121). Cells were observed daily under light microscope (Olympus-Japan). Cells with passage nos. from 10 to 20 were used in most of the experiments.

Four-week-old male mice C57BL/6J (16–18 g weight) were procured from an institutional animal care facility and acclimated for 1 week. The study was duly approved by the institutional animal ethics committee (IAEC). Mice were divided into three groups (n = 7). Group 1: Control group was given 1 million B16F10 melanoma cells subcutaneously into the ventral area in Matrigel and Vascular endothelial growth factor (VEGF), Group 2: B16F10 cells + NPC-402 treatment, Group 3: B16F10 cells + dacarbazine treatment. NPC-402 was administered intraperitoneally (i.p.) at 10 mg/kg for 5 days in a week by preparing as a suspension in transketolase, while the dacarbazine standard group was administered Dacarbazine at 60 mg/kg as a suspension in transketolase at alternate intervals thrice a week. The study was scheduled for a week. All of the mice were separately caged at 24°C ± 2°C, having free access to food with 12-h light/dark cycle. Weekly weight patterns were monitored, and mice were sacrificed by CO_2_ euthanasia. Blood was collected by cardiac puncture and subjected to centrifugation, and serum was stored at -80°C in the Matrigel plug assay.

### Cell viability assay

Enzymatic reduction of 3-[4,5-dimethylthiazole-2-yl]-2,5-diphenyltetrazolium bromide (MTT) by metabolic active cells to MTT–formazan by mitochondrial enzyme succinate dehydrogenase was used for the evaluation of cell viability as described earlier (23, 24). Briefly, after cell culture and followed by treatment with the test compound, the cells were further incubated for 24 h. The absorbance was recorded by a Multiskan plate reader (Thermo Electron Corporation) at 570 nm.

### Colony formation assay

B16F10 cells were seeded in six-well plates (250–300 cells/well). After 24 h, cells were subjected to treatment with different concentrations of NPC-402 for 24 h and further cultured for 5–7 days. Cells were fixed with 70% methanol and stained with crystal violet 0.1%. Stained colonies were captured by EVOS-FL Cell Imaging System (Thermo Fisher).

### Scratch assay (cell migration)

The scratch assay was performed as described previously (25). Briefly, cell proliferation was inactivated by mitomycin-C, and then cells were wounded by a microtip and washed with PBS. Cells were treated with the required concentrations of NPC-402 and further supplemented with fresh medium with or without VEGF (20 ng/ml).

### Annexin V/propidium iodide staining

Apoptosis was investigated using FITC Annexin-V/Dead Cell Apoptosis Kit (Molecular Probes-Life Technologies, OR, USA) as per protocol provided by the manufacturer. Stained samples were analyzed using BD FACS Calibur Aria flow cytometry.

### DNA fragmentation assay

Analysis of DNA fragmentation was performed as described previously (26). Briefly, after the treatment schedule, B16F10 cells were collected and washed with ice-cold 0.1 M Ethylenediamine tetra acetic acid (EDTA)-DPBS buffer and lysed in DNA lysis buffer and further incubated at 37°C for 1.5 h in a shaker water bath. After centrifugation, the supernatant was treated with proteinase K (250 μg/ml) at 50°C for an additional 60 min. DNA was extracted in phenol-chloroform and precipitated with ethanol and analyzed electrophoretically.

### Reactive oxygen species measurement *via* fluorescence microscopy/flow cytometry

Dichlorofluorescin diacetate (H_2_DCFDA) was used for the measurement of ROS generation as described previously (27). After completion of the treatment schedule, the dishes were incubated with H_2_DCFDA (5 μM, Sigma-Aldrich) for 15 min then followed by DPBS washing, and images were captured on fluorescent microscope on GPF-filter at ×20 resolution. Camptothecin (5 μM) was used as a positive control for the indicated time points (**Figures 4A, B**). Fluorescence of the images was quantified by using ImageJ software as reported earlier (28). We have also evaluated the ROS generation by flow cytometry (BD FACS Calibur Aria) by looking for the change in fluorescence due to the production of 2’, 7’-dichlorofluorescein (DCF). H_2_O_2_ (100 μM) was used as a positive control. B16F10 cells were exposed to H_2_O_2_ (100 μM) for 15 min prior to analysis.

### Lipid peroxidation assay

Lipid peroxidation was measured by quantifying malondialdehyde (MDA-Thiobarbituric acid (TBA) adducts) by a commercially available colorimetric assay (Sigma-Aldrich) as per the manufacturer’s protocol. Briefly, after termination of the experiment, cells were collected in 1.5-ml centrifuge tubes and homogenized on ice in MDA lysis buffer containing butylated hydroxytoluene (BHT) and then centrifuged at 13,000 g for 10 min to remove the insoluble material. The TBA solution was added to each Sample & Standard tube and incubated at 95°C for 60 min in a water bath and allowed to cool on an ice bath for 10 min. Finally, absorbance was documented at 532 nm by a spectrometer (Multiskan Spectrum plate reader, Thermo Electron Corporation).

### Study of mitochondrial membrane potential using fluorescence microscopy

Mitochondrial membrane potential (ΔΨm) was analyzed by JC-9 staining as described previously (28). Briefly, cells were grown on coverslips, and after attaining confluency of 60%–70%, the cells were treated with the indicated concentrations of NPC-402. After completion of treatment, JC-9 (5 μg/ml) dye in DPBS was given to cells and incubated for 25 min in culture conditions. After slide preparation, imaging was performed using EVOS-FL Microscope (Thermo Fisher) using red and green filters, and the images were quantified using the ImageJ software (28).

### Immunocytochemistry assay

Immunocytochemistry (ICC) experiments were carried out as reported previously (22, 28). Briefly, cells were incubated on coverslips in 35-mm culture dishes. After the completion of the treatment schedule, fixation of cells was carried out with 4% paraformaldehyde for 10 min at Room Temperature (RT). Cells were further permeabilized with 0.1% Triton X-100 for 10 min at RT. After blocking in 1% bovine serum albumin (BSA), cells were incubated with primary antibody overnight at 4°C. After proper washing with DPBS-T (Tween 20 0.1%), appropriate secondary antibodies were added to cells and incubated in the dark for 2 h at RT. Cells were mounted with ProLong Gold antifade reagent (4′,6-diamidino-2-phenylindole) (DAPI), and images were taken on EVOS-FL Cell Imaging System at ×20.

### Small interference RNA-mediated knockdown assay

Validated CHOP small interference RNA (siRNA) was procured from Santa Cruz Biotechnology, USA. Lipofectamine (Invitrogen, Life Technologies) and CHOP siRNA were diluted into opti-MEM 1 reduced serum medium (Gibco, Life Technologies) as per manufacturer’s instruction. B16F10 cells were incubated with a transfection mixture for 16 h at a final siRNA concentration of 50 pmol and supplemented with fresh medium. The cells were further subjected to treatment of NPC-402 as per experimental design.

### Cytochrome C release from mitochondrial membrane imaging assay

Cells were grown in coverslips overnight. Later, the cells were treated with/without NPC-402 for 12 h and then incubated with MitoTracker-DND-red (250 nM) for 30 min. The cells were washed thrice with warm DPBS followed by other steps in the ICC protocol by looking for the localization of cytochrome C as described.

### Preparation of cell lysates for western blotting

Cell lysates for protein expression studies were prepared as per our previous reports (29, 30). Briefly, after cell harvesting, cells were lysed with Radioimmunoprecipitation assay buffer (RIPA buffer) and protein quantification of the samples was measured by the Bradford colorimetric assay (Sigma-Aldrich) using BSA as standard. Equal amounts of protein (40 µg) were loaded to polyacrylamide gel, and the proteins were resolved in 8%–15% gel using Miniprotein Tetra System (Bio-Rad). The gel was transferred to Polyvinylidene fluoride (PVDF) membranes and immunoblotted using specific antibodies against various proteins. Secondary antibodies (Horseradish peroxidase (HRP)-conjugated) at a dilution of 1:2,000 (Santa Cruz/CST-USA) and directed against primary antibodies were used, and the blots were developed with Immobilon Western Chemiluminescent HRP Substrate. Signal intensities of bands were detected and quantified by ChemiDoc Imaging System (Bio-Rad).

### Matrigel plug assay

The assay was performed as described previously (31). Briefly, 0.5 ml of Matrigel and VEGF (100 ng) with or without B16F10 melanoma cells (1 million cells/animal) were injected subcutaneously into the ventral area of C57BL/6J mice. Animals of group 2 were dosed daily with NPC-402 (10 mg/kg/body weight; i.p./oral) and animals of group 3 were dosed with standard drug dacarbazine at alternate intervals (60 mg/kg/body weight; i.p./oral) for 1 week. On day 7, animals were sacrificed, and intact Matrigel plugs were removed and photographed showing the extent of vascularization. Matrigel plugs were analyzed and quantified for neovascularization by measuring their size and hemoglobin (Hb) content using the Drabkin reagent.

### Statistical analysis

Data are expressed as the mean ± standard deviation (SD). INSTANT statistical software was used to perform statistical analysis. Data are presented as mean ± SD from three independent experiments. Comparison between two groups was performed by Student’s t-test and that among groups was carried out by one-way ANOVA for statistical significance. p ≤ 0.05 was considered statistically significant.

## Results

### Screening of *O*-Alkylated/Benzylated Glycyrrhetinic acid analogs against melanoma

Eighteen different analogs were synthesized from glycyrrhizin through GA at the C3 position (**Figure S1A**). After the cell-based screening assay among the different analogs (**Figure S1B**), 3-(3-methyl-but-2-enyloxy)-11-oxo-olean-12-ene-29-oic acid (NPC-402) was found most potent against B16F10 melanoma cells with an IC_50_ of 16 µM. The yield percentage of NPC-402 obtained was 90% with a purity of 98.7% analyzed from HPLC-chromatogram *t*_R_ = 48.4 min (98% purity); yield: 90%; IR (CHCl_3_): ν_max_ 3,441, 2,927, 2,867, 1,724, 1,658, 1,658, 1,619, 1,454, 1,385, 1,327, 1,310, 1,257, 1,210, 1,152, 1,084, and 1,046 cm^-1^ (**Figure S1D**). **Figure S1C** describes the ^1^H NMR (400 MHz, CDCl_3_): *δ* (ppm) 5.63 (s, 1H, CH-12), 5.34 (t, 1H, CH-2´), 4.62 (m, 2H, CH_2_-1´), 3.23 (dd, 1H, CH-2), 2.81 (tt, 1H, CH-18), 2.34 (s, 1H, CH-9), 1.77 (s, 3H, CH-3´), 1.72 (s, 3H, CH-4´), 1.36 (s, 3H, Me-27), 1.26 (s, 3H, Me-25), 1.14 (s, 3H, Me-29), 1.13 (s, 3H, Me-26), 1.01 (s, 3H, Me-23), 0.81 (s, 3H, Me-24), 0.80 (s, 3H, Me-28), 0.71 (d, 1H, CH-5); HR-ESIMS: *m/z* 539.4106 [M+H]^+^ calculated for C_35_H_54_O_4_ + H^+^ (539.4094).

### NPC-402 shows potent growth inhibition in melanoma cells

**Figure 1:**
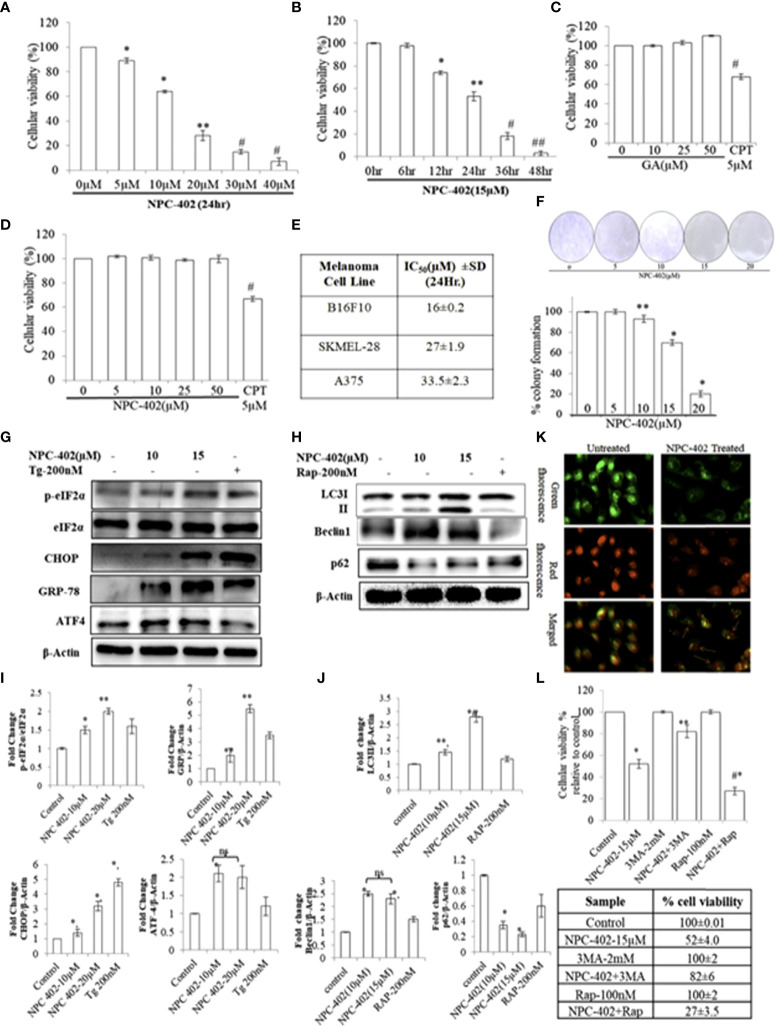
NPC-402 Shows Potent Growth Inhibition and ER stress-mediated autophagy induction in B16F10 melanoma cells. **(A)** Bar graph represents the cell viability analysis of B16F10 cells treated with/without NPC-402 for 24 h using the MTT assay (*represents *p* < 0.001, **p < 0.01, ^#^ *p* < 0.05, control vs. NPC-402 treatment). **(B)** Bar graph represents the cell viability analysis of B16F10 cells treated with 15 µM of NPC-402 for different time intervals using the MTT assay (*represents *p* < 0.001, **p < 0.01, ^#^ *p* < 0.05, control vs. NPC-402 treatment). **(C)** Bar graph represents the cell viability analysis of B16F10 cells treated with/without glycyrrhetinic acid (GA) for 24 h using the MTT assay. Camptothecin (CPT) serves as a positive control (^#^ *p* < 0.05, control vs. CPT treatment). **(D)** Bar graph represents the cell viability analysis of HaCaT cells treated with/without NPC-402 for 24 h using the MTT assay. Camptothecin (CPT) serves as a positive control (^#^ *p* < 0.001, control vs. CPT treatment). **(E)** Represents the IC50 of NPC-402 treatment in penal of melanoma cells. **(F)** The diagram represents the colony formation in NPC-402-treated B16F10 cells, and the bar graph represents the percentage colony formation in B16F10 cells treated with/without NPC-402 (*p < 0.00, **p < 0.01, control vs. NPC-402 treatment). Results are representative of three similar experiments. **(G)** Western blot represents the protein expression of p-eIf2α, eIf2α, CHOP, GRP-78, and ATF4 using beta-actin as a loading control in B16F10 cells treated with NPC-402. **(I)** Bar graph represents the densitometry analysis of protein in fold change p-eIf2α/eIf2α, CHOP, GRP-78, and ATF4 using beta- actin as a loading control in B16F10 cells treated with NPC-402 using Image Lab™ Software Version 3.0 (Bio-Rad). **(H)** Western blot represents the protein expression of LC3II, Beclin1, and P62 using beta-actin as a loading control in B16F10 cells treated with NPC-402. **(J)** Bar graph represents the densitometry analysis of protein in fold change of LC3II, Beclin1, and P62 using beta-actin as a loading control in B16F10 cells treated with NPC-402 using Image Lab™ Software Version 3.0 (Bio-Rad). **(K)** Microscopic images represent the detection of autolysosomes thru acridine orange staining in NPC-402-treated B16F10 cells. **(L)** The bar graph and table represent the cell viability analysis of B16F10 cells treated with/without NPC-402, 3MA, or Rap or in combinations for 24 h using the MTT assay (*represents *p* < 0.001 control vs. NPC-402, #*p* < 0.001 control vs. NPC-402+3MA) (*p < 0.001, **p < 0.01, control vs. NPC-402 treatment). ns, non-significant.

The cytotoxic effect of NPC-402 was first investigated at various concentrations on different melanoma cell lines (B16F10, A375, and SKMEL-28) and normal cell lines (human immortalized keratinocyte HaCaT). Upon treatment of B16F10 cells with NPC-402, cellular viability decreases significantly and dose-dependently (**Figure 1A**). We calculated the IC_50_ of NPC-402 treatment of melanoma cells at 24 h and found it to be 16 ± 0.2 μM for B16F10, 27 ± 1.9 μM for A375, and 33.5 ± 2.3 μM for SKMEL-28 (**Figure 1E**). B16F10 cells were treated with 15-μM concentration of NPC-402 at different time intervals, and NPC-402 induces a significant cell death effect in a time-dependent manner, and we achieved 96% ± 2% cell death 48 h posttreatment (**Figure 1B**). We have also checked the effect of parent compound GA on B16F10 cells and found that it did not alter cell growth. Camptothecin 5 μM was used as a standard positive cytotoxic control (**Figure 1C**). In contrast, NPC-402 shows no significant cytotoxic effect on human immortalized keratinocyte cell line HaCaT up to a concentration >50 μM (**Figure 1D**), indicating the specificity of NPC-402 for melanoma cells over normal cells. Furthermore, we performed colony formation assay and found that NPC-402 significantly decreases cell colony formation at higher concentrations (**Figure 1F**). The percentage of colony formation clearly reveals that NPC-402 at lower concentrations was cytostatic whereas NPC-402 at higher concentrations was cytotoxic (**Figure 1F**).

These results cite that the effectiveness of NPC-402 in inducing toxicity is cell type-specific and indicate that NPC-402 is capable of eliciting different cellular responses that need comprehensive characterization to fully understand how it can be taken forward as a potential anti-melanoma candidate drug.

### NPC-402 leads to endoplasmic reticulum stress-mediated autophagy induction that culminates into apoptosis in B16F10 melanoma cells

To investigate the effect of NPC-402 on ER stress response, we evaluated the expression levels of key proteins related to ER stress: p-eIF2α, GRP78, CHOP, and ATF4. We found that NPC-402 at 12 h posttreatment significantly upregulates the expression of ER stress response proteins (**Figures 1G, I**), indicating that NPC-402 leads to the induction of ER stress. Thapsigargin (Tg; 200 nM) was used as an ER stress positive control. The exact role of autophagy in melanoma is not yet known, but research reports suggest that ER stress triggers autophagy induction. We investigated whether NPC-402 has any effect on cellular autophagy response by looking for the expression profile of autophagy-related proteins LC3I/II, Beclin1, and p62. We observed an elevated expression level of LC3-II by calculating the conversion ratio of LC3-I to LC3-II after NPC-402 treatment of cells (**Figures 1H, J**). The expression level of Beclin1 is upregulated significantly compared to that of control (**Figures 1H, J**). In addition, we observed downregulation of p62 in NPC-402-treated cells, being an essential protein in the autophagy process (**Figures 1H, J**). Furthermore, we looked for autolysosomes in NPC-402-treated B16F10 cells, and acridine orange staining revealed an increase in the volume of acidic vesicular organelles (AVOs) that indicates the autophagy response (**Figure 1K**). All of these findings indicate the induction of autophagy by NPC-402 treatment in B16F10 cells. To confirm the cell death role of autophagy in NPC-402-treated B16F10 cells, cell viability analysis was performed and autophagy inducer rapamycin (Rap) and autophagy inhibitor 3-methyladenine (3MA) were used as respective controls. We found that NPC-402 (at 15 µM) alone induces 48% cell death compared to control, whereas NPC-402+3MA (2 mM) induce 18% cell death compared to control and NPC-402+Rap (100 nM) significantly induce 73% cell death compared to control (**Figure 1L**). From the above results, we conclude that inhibition of autophagy by 3MA blocks NPC-402-induced cell death and activation of autophagy by Rap enhances NPC-402-induced cell death and confirm that the nature of autophagy induced by exposure to NPC-402 of B16F10 cells has a cytotoxic effect.

### Chemical chaperone 4-phenylbutyric acid interferes with NPC-402-induced apoptosis *via* relieving endoplasmic reticulum stress-mediated autophagy induction in B16F10 melanoma cells

**Figure 2:**
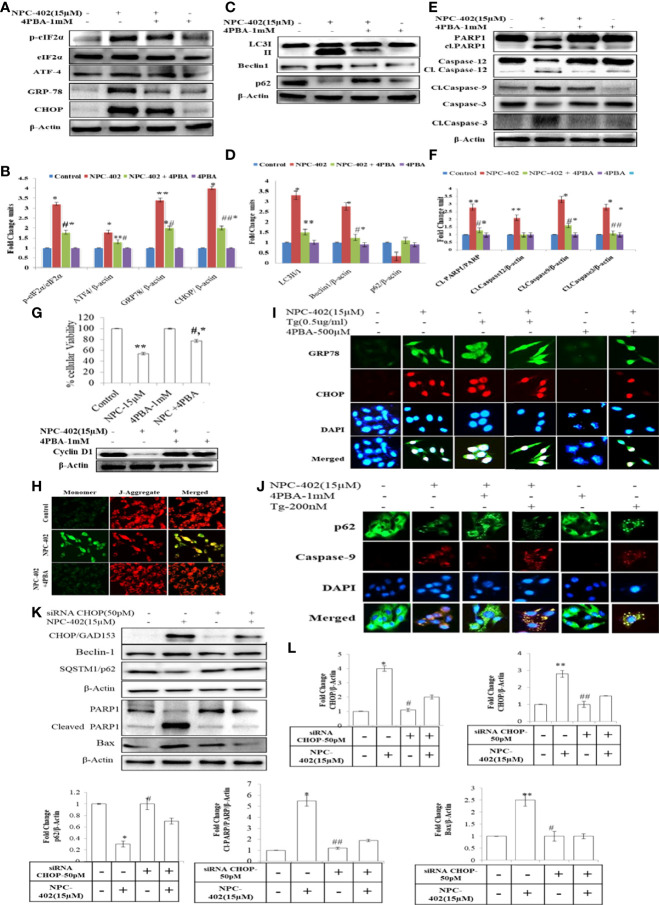
4PBA interferes with NPC-402-induced ER stress response and autophagy in B16F10 cells. **(A)** Represents the Western blot of protein expression of p-eIf2α, eIf2α, CHOP, GRP-78, and ATF4 using beta-actin as a loading control in B16F10 cells treated with NPC-402 along with/without 4PBA. **(B)** Bar graph represents the densitometry analysis of ER stress proteins in fold change in comparison to beta-actin in B16F10 cells treated with NPC-402 along with/without 4PBA using Image Lab™ Software Version 3.0 (Bio-Rad). **(C)** Represents the Western blot of protein expression of LC3I/II, Beclin1, and p62 using beta-actin as a loading control in B16F10 cells treated with NPC-402 along with/without 4PBA. **(D)** Bar graph represents the densitometry analysis of autophagy proteins in fold change in comparison to beta-actin in B16F10 cells treated with NPC-402 along with/without 4PBA using Image Lab™ Software Version 3.0 (Bio-Rad). **(E)** Represents the Western blot of protein expression of PARP1, caspase-12, caspase-9, and caspase-3 using beta-actin as a loading control in B16F10 cells treated with NPC-402 along with/without 4PBA. **(F)** Bar graph represents the densitometry analysis of apoptosis proteins in fold change in comparison to beta-actin in B16F10 cells treated with NPC-402 along with/without 4PBA using Image Lab™ Software Version 3.0 (Bio-Rad)**. (G)** Bar graph represents cell viability percentage of B16F10 cells treated with NPC-402 along with/without 4PBA. Western blot represents the protein expression of cyclin D1 in B16F10 cells treated with NPC-402 along with/without 4PBA. **(H)** Microscopic images represent the JC-9 staining for the detection of ΔΨm in B16F10 cells treated with/without NPC-402 along with/without 4PBA. **(I)** ICC images of GRP-78 (green), CHOP (red), costained with DAPI (blue) on B16F10 treated with NPC-402 along with/without 4PBA. The images were taken at ×40 under microscope (EVOS). **(J)** ICC images of p62-78 (green) cleaved caspase-9 (red) costained with DAPI (blue), on B16F10 treated with NPC-402 along with/without 4PBA. The images were taken at ×40 under microscope (EVOS). **(K)** Represents the Western blot of protein expression of CHOP, Beclin1, p62, caspase-12, caspase-9, and caspase-3 using beta-actin as a loading control in B16F10 cells treated with NPC-402 along with/without 4PBA. **(L)** The bar graphs represents the fold change expression of proteins evaluated by densitometry method using Image Lab™ Software Version 3.0 (Bio-Rad) (*p < 0.01, **p < 0.05 for control vs. NPC-402 and ^#^ *p* < 0.01, ^##^ *p* < 00.05 for NCP-402 vs. NPC-402+4PBA).

To investigate whether NPC-402 exposure of melanoma B16F10 cells leads to ER stress-mediated autophagy induction and apoptosis, we employed chemical chaperone 4-phenylbutyric acid (4PBA) and evaluated its cytoprotective nature upon treatment of B16F10 cells with NPC-402 as it rescues cells from ER stress effects. 4PBA is a well-known attenuator of ER stress/UPR used in different models of mammalian cell cultures and has great clinical potential in restoring ER stress-disturbed homeostasis (32). 4PBA treatment was given 2 h prior to NPC-402 in B16F10 cells. We found significant downregulation in ER stress-related proteins p-eIF2α, CHOP/GAD153, and GRP78 after cotreatment of B16F10 cells with NPC-402 and 4PBA compared to NPC-402 only treatment (**Figures 2A, B**), indicating that 4PBA attenuates NPC-402-induced ER stress response. We also found that attenuation of ER stress by 4PBA leads to inhibition of NPC-402-induced autophagy by observing significant downregulation of Beclin1 and accumulation of p62 after cotreatment of B16F10 cells with 4PBA+NPC-402 compared to NPC-402 only treatment (**Figures 2C, D**). This confirms that NPC-402 exposure of melanoma cells induces an ER stress response that leads to the induction of autophagy. We also tested whether 4PBA interferes with NPC-402-induced apoptosis and found a significant decrease in cell death rate after cotreatment of B16F10 cells with 4PBA+NPC-402 compared to NPC-402 only treatment, demonstrating that relieving ER stress by 4PBA attenuates the apoptotic effect of NPC-402 (**Figure 2G**). We also observed significant downregulation in the expression level of caspase-12, cleaved caspase-3, and caspase-9 in these cells cotreated with 4PBA+NPC-402 (**Figures 2E, F**). NPC-402 also leads to inhibition of cyclin D1 in B16F10 cells, depicting cell cycle arrest at G1-stage that promotes apoptosis, and restoration of cyclin D1 in cotreated (4PBA+NPC-402) conditions releases cells from G1-stage and promotes cell proliferation (**Figure 2G**). These results suggest an indispensable role of NPC-402-mediated ER stress response in regulating caspase-dependent apoptosis signaling *via* autophagy in B16F10 cells. We further investigated the molecular mechanism of NPC-402-induced cell death effect *via* ER stress and autophagy. We knocked down the expression of CHOP, as it is known to regulate autophagy by increasing the expression of autophagy-related proteins by using 50 pM concentration of CHOP siRNA and analyzed apoptosis. We found that Beclin1 is significantly decreased and the expression of SQSTM/p62 is increased in siRNA-transfected cells treated with NPC-402 compared to NPC-402 only treatment (**Figures 2K, L**). Blockage of NPC-402-induced ER stress by silencing CHOP prevents cell death in B16F10 cells as is clear from the expression of cleaved Parp1 and Bax (**Figures 4K, 4K.d, 4K.e**). ICC of CHOP and GRP78 confirms our finding (**Figure 2I**), and the results of p62 and cleaved caspase-9 were also confirmed by ICC (**Figure 2J**). We have also verified that 4PBA treatment of B16F10 cells alleviates the loss of ΔΨm analyzed *via* rhodamine-123 staining (**Figure 2H**). ER stress results in accumulation of ROS causing oxidative stress and also upregulates CHOP that plays a great role in potentiating the oxidative stress-mediated cell death response (33).

### NPC-402 induces apoptosis in B16F10 cells

**Figure 3:**
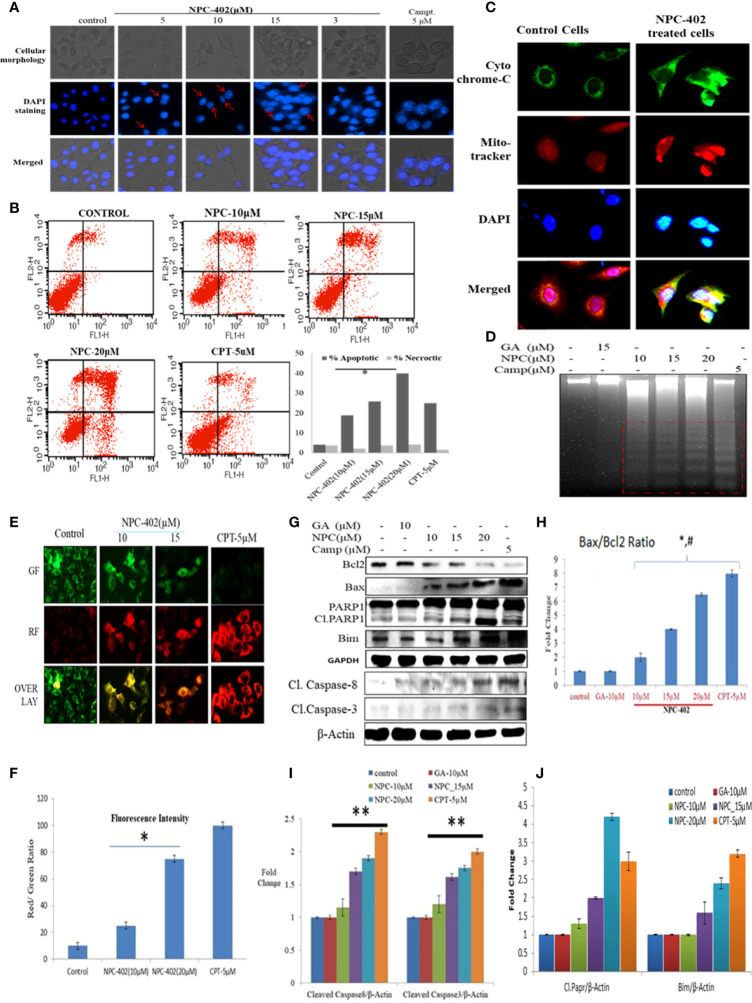
NPC-402 induces mitochondrial dysfunction and modulates the expression level of proapoptotic and antiapoptotic markers. **(A)** Staining of B16F10 cells with DAPI on treatment with NPC-402. Cellular morphology and nuclear fragmentation, indicated by red arrows, were seen in melanoma cell line post 24 h treatment, and camptothecin was used as a positive control. **(B)** Annexin V/PI FITC staining of B16F10 cells was done to find the apoptosis induction in B16F10 cells post 12 h treatment of NPC-402 and found increasing percentages of Annexin-V-bound cells. **(C)** Represents the confocal imaging for the detection of cytochrome C translation into cytosol upon exposure of B16F10 cells to NPC-402 treatment for 12 h compared with untreated cells. **(D)** Gel diagram represents DNA fragmentation in B16F10 cells treated with/without NPC-402 for 24 h, camptothecin as a positive control, and GA as a parent molecule of NPC-402. **(E)** Microscopic images represent the JC-9 staining for the detection of ΔΨm in B16F10 cells treated with/without NPC-402; CPT acts as a positive control. **(F)** Red/green fluorescences of panel **(B)** were quantified by using ImageJ software (*p < 0.001; control vs. NPC-402 treatment). **(G)** Represents the Western blots of Bcl2, Bax, Parp1, Bim, caspase-8, and caspase-3 in B16F10 cells treated with NPC-402 for 24 h. β-Actin was used as a loading control. **(H)** Bar graphs represent the densitometry analysis of Bax/Bcl_2_ ratio in B16F10 cells treated with NPC-402 for 24 h using Image Lab™ Software Version 3.0 (Bio-Rad). **(I)** Bar graphs represent the densitometry analysis of cleaved caspase-8 and cleaved caspase-3 in B16F10 cells treated with NPC-402 for 24 h using Image Lab™ Software Version 3.0 (Bio-Rad). (**J**) Bar graphs represent the densitometry analysis of cleaved PARP1 and Bim in B16F10 cells treated with NPC-402 for 24 h (*p < 0.001, control vs. NPC-402 treatment; **p < 0.01, control vs. NPC-402 treatment; p<0.05 GA treated vs. NPC-402).

NPC-402 exposure of B16F10 cells in 24-h treatment induces morphological changes compared to control cells. These changes include surface detachment, cell shrinkage, and multinucleation, and increasing the concentration of NPC-402 causes disruptive changes in the nuclei of cells. Chromatin condensation was observed with significant nuclear shrinkage and fragmentation in treated cells compared to control (**Figure 3A**). We further evaluated the induction of apoptosis in B16F10 cells upon NPC-402 treatment through Annexin V-FITC staining by looking for inner membrane-bound phosphatidylserine (PS). NPC-402-treated B16F10 cells after 24 h significantly increased Annexin V-positive cells. Camptothecin was used as an experimental control (**Figure 3B**). Mitochondrial cytochrome C plays an important role in apoptosis *via* the apoptotic protease-activating factor (Apaf) and the release of cytochrome C from mitochondria to cytosol, leading to the cascade of events that leads to the activation of caspases that in turn leads to nuclear DNA fragmentation (34). Translocation of cytochrome C to cytosol (**Figure 3C**) in NPC-402-treated B16F10 cells was confirmed through confocal microscopy by using MitoTracker as shown in **Figure 3C**. We have also found that NPC-402 induces DNA fragmentation in B16F10 cells compared to untreated and GA-treated cells (**Figure 3D**). Rhodamine-123 was used to analyze the effect of NPC-402 on Δψm, and we found that NPC-402 leads to a significant loss of Δψm in a dose-dependent nature compared to untreated cells (**Figures 3E, F**). Loss of green fluorescence clearly indicates mitochondrial depolarization due to NPC-402 treatment of B16F10 cells. The activation of caspases, Bcl-2/Bax ratio, and other proteins associated with apoptosis were examined by Western blotting. Bax/Bcl-2 ratio acts as a rheostat that determines cellular susceptibility to apoptosis, and elevated levels of this ratio indicate apoptosis (35) and NPC-402 significantly increases the ratio of Bax/Bcl-2 dose-dependently compared to untreated cells (**Figures 3G, H**). Apoptosis induction by NPC-402 is also clear from increased levels of initiator cleaved caspase-8 and executor caspase-3 in a dose-dependent nature (**Figures 3G, I**). Cleaved Poly [ADP-ribose] polymerase 1 (PARP-1) is also significantly increased in NPC-402-treated cells compared to untreated cells (**Figures 3G, H**). Taken together, these results suggest that NPC-402 causes stable induction of apoptosis and decreases the expression level of proapoptotic proteins.

### NPC-402 induces oxidative stress in B16F10 cells

**Figure 4:**
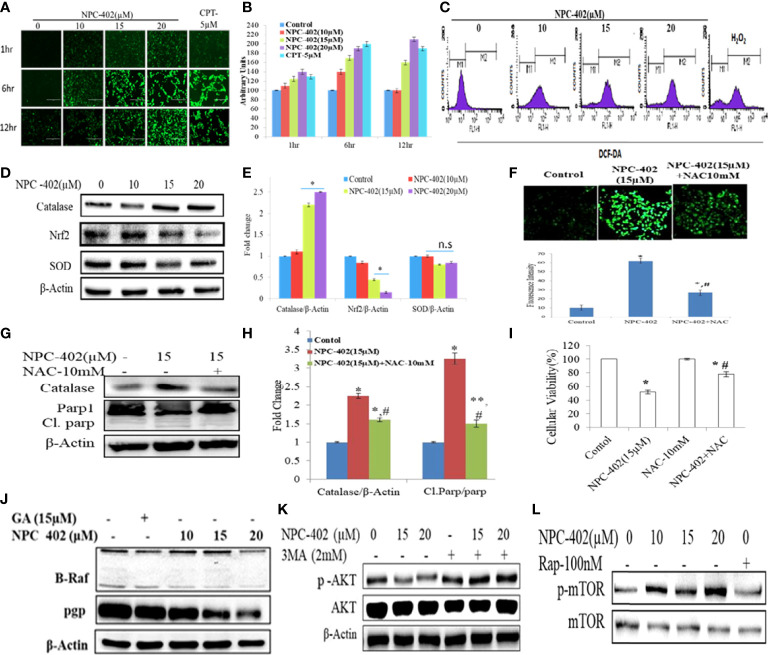
Induction of oxidative stress leads to apoptosis and inhibition of MAP kinase/AKT pathways in NPC-402-treated B16F10 cells. **(A)** Microscopic images represent time-dependent ROS fluorescence in B16F10 cells treated with/without NPC-402 by using H_2_DFDA dye. **(B)**. Bar graphs represent fluorescence intensity time- and dose-dependently in NPC-402-treated/without treatment B16F10 cells using ImageJ software. **(C)** Represents flow cytometric analysis of ROS generation dose-dependently in NPC-402-exposed B16F10 cells, and H_2_O_2_ was used as an experimental control. **(D)** Represents Western blots of protein SOD, catalase, and Nrf2 in B16F10 cells treated with NPC-402. **(E)** Bar graph represents the densitometry analysis of catalase, Nrf2, and SOD using Image Lab™ Software Version 3.0 (Bio-Rad). **(F)** Microscopic images represent the ROS fluorescence by H_2_DFCA staining in B16F10 cells treated with NPC-402 or NAC or both, and the bar graphs represent ROS quantification thru ImageJ software. **(G)** Immunoblots of catalase, Parp1 in B16F10 cells treated with NPC-402/NAC or both. β-Actin was used as a loading control. **(H)** Bar graph represents the densitometry analysis of catalase, Parp1 in B16F10 cells treated with NPC-402/NAC or both using Image Lab™ Software Version 3.0 (Bio-Rad). **(I)** Bar graph represents the cell viability analysis of B16F10 cells treated with/without NPC-402 or NAC for 24 h using the MTT assay (*p < 0.001 control vs. NPC-402, ^#^ *p* < 0.001, NPC-402 vs. NPC-402+NAC) (*p < 0.001, control vs. NPC-402 treatment, **p < 0.01, control vs. NPC-402 treatment, ^#^ *p* < 0.001, control vs. NAC treatment). **(J)** Immunoblot represents the protein expression of B-RAF and PGP using β-actin as a loading control in B16F10 cells treated with NPC-402. **(K)** Immunoblot represents the protein expression of p-AKT/AKT using β-actin as a loading control in B16F10 cells treated with NPC-402 and 3MA. **(L)** Immunoblots represent the protein expression of p-mTOR/mTOR using β-actin as a loading control in B16F10 cells treated with NPC-402. Rapamycin serves as a positive control (*p < 0.001, **p <0.01, control vs. NCP402 treatment).

To investigate the effect of NPC-402 on ROS generation, DCFH-DA-based flow cytometry and fluorescent imaging analysis were performed to study oxidative stress response. We detected a significant elevation of intracellular ROS in NPC-402-treated B16F10 cells time- and dose-dependently compared to untreated cells (**Figures 4A, B**), and the same results were obtained in cytometry analysis, as NPC-402-treated samples show increased area under the curve of FL-2 peak (**Figure 4C**). Furthermore, we analyzed the potential of NPC-402 to induce oxidative stress by measuring the MDA levels in NPC-402-treated B16F10 cells, and we observed that NPC-402 dose-dependently increased the lipid peroxidation levels as compared to that of control (**Figure S3**). To confirm our microscopy findings, we additionally performed Western blotting analysis of key oxidative stress-related proteins catalase, Nrf2, and SOD and found that NPC-402 increases the expression of catalase and decreases the expression of Nrf2 in a dose-dependent manner (**Figures 4D, E**). This disturbance related to the change in the expression of oxidative marker proteins leads to the induction of apoptosis in B16F10 melanoma cells. N-acetylcysteine (NAC) is a potent quencher of ROS, and here, NAC 10 mM was used to quench the NPC-402-induced ROS in B16F10 cells. We found that NAC significantly alleviates ROS generation, restores catalase expression, and blocks activation of cleaved PARP-1 in cotreated (NAC+NPC-402) B16F10 cells compared to NPC-402 only treatment (**Figures 4F–I**).

### NPC- 402 attenuates MAPK/AKT signaling pathway in B16F10 melanoma cells

The MAPK pathway is activated in all melanomas, regulating cell survival, proliferation, and metastasis (36). We studied the effect of NPC-402 on MAPK signaling in B16F10 melanoma cells by Western blotting and found that NPC-402 treatment decreases the expression of phospho-MEK, phospho-ERK (**Figure 4J**, **Figure S2A**), and P-AKT (**Figure 4K**, **Figure S2B**) significantly in a dose-dependent nature. The effect of NPC-402 on stress kinases was explored, and it was found that NPC-402 downregulates the expression of phospho-P38 dose-dependently. However, NPC-402 significantly increases the expression in phosphorylation of p-JNK (**Figures S2D–S2I**). It has been reported that p-JNK induces cell death *via* c-Jun/cyclin D1 axis and plays a key role in ER stress-mediated autophagy induction. In 60% of melanoma cases, the primary cause is BRAF mutation, thus we analyzed the effect of NPC-402 on BRAF expression and observed that BRAF is significantly downregulated by 3-fold at higher concentrations (**Figure 4J**, **Figure S2A**). In addition, we also checked the PGP protein that is related to drug efflux in case of cancer cells and is normally active to efflux out the drugs taken in by cells in defense response. NPC-402 was found to reduce PGP levels (**Figure 4J**, **Figure S2A**), suggesting that the drug effusion property of B16F10 cells is reduced drastically, inducing apoptosis-related events. We further checked for the expression of p-mTOR and p-AKT through Western blotting analysis and found that NPC-402 treatment induces the expression of p-mTOR but downregulates p-AKT (**Figures 4K, L**), suggesting that the cell death potential of NPC-402 is independent of mTOR but is executed through involvement of AKT. Overall, these results suggest that NPC-402 treatment inhibits MAPK family proteins toward potentiating ER stress-mediated apoptosis.

### NPC-402 inhibits VEGF-induced tumor angiogenesis in the Matrigel plug assay

**Figure 5:**
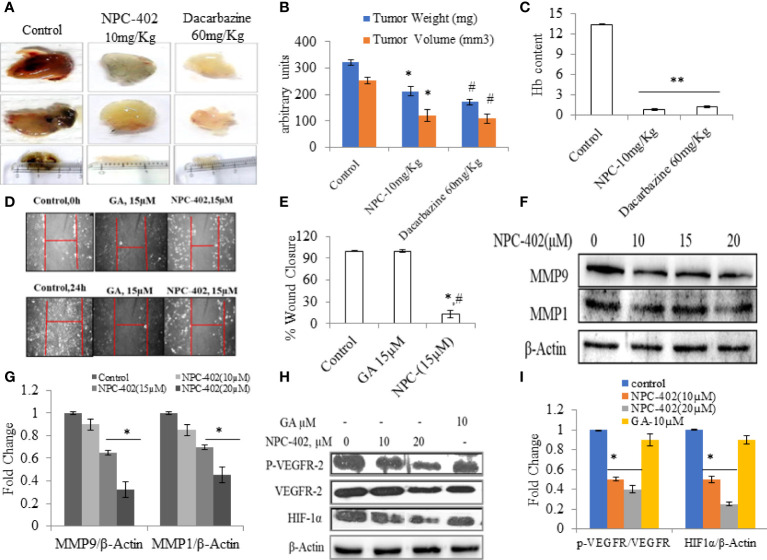
NPC-402 inhibits VEGF-mediated tumor angiogenesis in Matrigel assay. **(A)** Represents *in vivo* angiogenesis *via* Matrigel assay in C57BL/6J mice treated with/without NPC-402, and dacarbazine was used as a standard drug against melanoma. **(B)** Bar graph represents the tumor weight and volume of C57BL/6J mice treated with/without NPC-402 (*p < 0.001 control vs. NPC-402 group, *^#^p* < 0.001 control vs. dacarbazine group). **(C)** Bar graph represents Hb content of tumors, C57BL/6J mice treated with/without NPC-402 (*p < 0.001 control vs. NPC-402 group, *^#^p* < 0.001 control vs. dacarbazine group). **(D)** Images represent the cell migration assay (*in vitro*) in B16F10 cells treated with NPC-402. **(E)** Bar graphs describes the percentage wound closure in B16F10 cells treated with/without NPC or GA (*p < 0.001 control vs. NPC-402, *^#^p* < 0.001 GA vs. NPC-402). **(F)** Represents the Western blots of MMP1 and MMP9 in B16F10 cells treated with NPC-402 for 24-h. β-Actin was used as a loading control. **(G)** Bar graphs represent the densitometry analysis of MMP9 and MMP1 in B16F10 cells treated with NPC-402 for 24 h using Image Lab™ Software Version 3.0 (Bio-Rad). **(H)** Represents the Western blots of p-VEGFR2, VEGFR2, and HIF1α in B16F10 cells treated with NPC-402 for 24 h. β-Actin was used as a loading control. **(I)** Bar graphs represent the densitometry analysis of p-VEGFR2, VEGFR2, and HIF1α in B16F10 cells treated with NPC-402 for 24 h using Image Lab™ Software Version 3.0 (Bio-Rad) (*p < 0.001, control vs. NPC-402 treatment; **p < 0.01, control vs. NPC-402 treatment).

Angiogenesis is the key stride in tumor metastasis (37). We analyzed the effects of NPC-402 on VEGF-induced angiogenesis *in vivo*. C57BL/6J mice were dosed i.p. with NPC-402 (10 mg/kg/body weight) after implantation of Matrigel with or without B16F10 cells. The Matrigel plug of the control group revealed a marked increase in vascularization and tumor size as is evident from deep red appearance and high Hb content (**Figures 5A, C**). Matrigel plugs of animals dosed with NPC-402 (10 mg/kg/body weight) and dacarbazine (60 mg/kg/body weight) show significant alleviation in vascularization, tumor volume, and weight and appear white with negligible Hb content (**Figures 5A, B**), indicating that NPC-402 significantly inhibited angiogenesis. Furthermore, we also investigated Vascular endothelial growth factor receptor 2 (VEGFR2) and Hypoxia-inducible factor 1-alpha (HIF1α) protein expression that activates genes involved in migration, angiogenesis, and survival, such as VEGFs that are found to be overexpressed in tumor cells, suggesting that these proteins also have involvement in tumor progression (38). We found that NPC-402 reduces the expression of these proteins (**Figures 5H, I**), suggesting that NPC-402 alters the signaling response of melanoma cells that is associated with growth, development, and migration of the tumor. In addition, we performed a wound healing assay and found that NPC-402 significantly reduces the wound-healing ability of B16F10 cells as compared to that of control (**Figures 5D, E**). In melanoma, upregulation of matrix metalloproteinases (MMPs) is associated with migration of the tumor, and upon treatment of B16F10 cells with NPC-402, it dose-dependently downregulates the expression of MMP1 and MMP9 compared to untreated (**Figures 5F, G**). Similar results were obtained in dacarbazine-treated mice that was used as a positive control and is a standard anticancer drug used against melanoma treatment.

## Discussion

Cancer is generally defined as a malignant process of autonomous uncontrolled cell proliferation with the ability to metastasize and resistant to a normal cell death program. Cancer is the second leading cause of death in the world—only surpassed by cardiovascular diseases. Natural products have been widely used for their chemopreventive potential by interfering with cancer morbidities, but lately, drug resistance has become a challenge in cancer chemoprevention, so there is a need for discovering new chemical entities (NCEs) to overcome the burden of cancer pathologies. Among biologically beneficial organic compounds synthesized by plants, pentacyclic triterpenoids have been extensively explored for their appealing pharmacological properties. However, due to poor biological effects of pentacyclic triterpenoids on their molecular targets, these natural products have been used as important templates for the preparation of more active derivatives.

The 18β-glycyrrhetinic acid, a pharmacologically active secondary metabolite from a medicinally reputed plant *G. glabra*, shows significant cytotoxic and antitumor properties. The molecular mechanisms and proapoptotic targets of 18β-glycyrrhetinic acid have been exhaustively explored over the last decade. With the advantage of being an easily available and inexpensive triterpene, a large number of analogs have been synthesized on the basis of 18β-glycyrrhetinic acid scaffold with IC_50_ <30 μM.

In the present study, we have explored the therapeutic efficacy and molecular mechanism of the anti-melanoma effect of NPC-402 [3-(3-methyl-but-2-enyloxy)-11-oxo-olean-12-ene-29-oic acid], a triterpenoid ether derivative of GA (**Figures S1A–S1D**) on melanoma cells (B16F10) with only marginal toxicity on normal cells. In a cancer therapeutic perspective, it is indispensable for chemotherapeutics to kill cancerous lesions with minimum toxic manifestations on normal tissues (39). Therefore, human keratinocytes (HaCaT cells) were used as a reference model of normal or non-tumorigenic skin cells. We found that NPC-402 is non-toxic up to a concentration of 50 μM in HaCaT cells in 24-h treatment (**Figure 1D**) compared to B16F10 cells with an IC_50_ at 16 µM (**Figure 1A**). Moreover, NPC-402 was found to induce cell death in a time-dependent manner (**Figure 1B**). GA, the parent molecule of NCP-402, was found to be devoid of toxic effects on B16F10 cells (**Figure 1C**). We tested the toxicity of NPC-402 on various melanoma cell line models and evaluated the IC_50_ as shown in **Figure 1E** and performed colony formation assay and found that NPC-402 acts as a cytostatic agent at lower concentrations and caused cytotoxicity at higher concentrations (**Figure 1F**).

We evaluated the effect of NPC-402 on ER stress. ER is a cytoplasm-based dynamic membrane-bound organelle that is responsible for protein folding and also involved in posttranslational modification, calcium storage, and lipid metabolism (10). Inimical conditions such as calcium depletion, truncated or misfolded proteins, and oxidative stress in the cell lead to a condition known as ER stress that leads to UPR *via* activating associated signaling pathways (10). Targeting ER stress is considered as the therapeutic future in melanoma treatment (40). In line with this, we observed that NPC-402 treatment induces ER stress response in cells as was evident by the increased expression of GRP78 that evokes the UPR and also halts protein translation *via* GRP78-eIF2α-CHOP signal transduction pathway (**Figures 1G, I**). Severe and prolonged ER stress deteriorates cellular physiology and switches cell fate toward apoptosis. Numerous studies have suggested that ER stress and autophagy are systematically interconnected, wherein the UPR and ER stress pathway stimulates the induction of basal autophagy that potentiates cell death. Induction of autophagy was observed upon NPC-402 treatment of melanoma cells corroborated by increased acidic vacuole formation (**Figure 1K**), LC3I/II lipidation, upregulation of Beclin1, and downregulation of cargo cum adapter protein SQSTM1/P62 (**Figures 1H, J**). In order to validate the role of autophagy in NPC-402-mediated cell death, the cell viability assay was performed in the presence of an autophagy activator, Rap, and an autophagy inhibitor, 3MA. The results clearly indicate that Rap potentiated, whereas 3MA mitigated, the cell death effects of NPC-402 (**Figure 1L**).

Chemical chaperone 4PBA is a noticeable molecule that mitigates the ER stress induced by hostile conditions that has been confirmed in our previous studies (41). We analyzed whether 4PBA interferes with NPC-402-induced apoptosis *via* relieving ER stress-mediated autophagy induction in B16F10 melanoma cells. We observed that 4PBA mitigates ER stress induced by NPC-402 evident from the expression of GRP78/Bip that in turn prevented downstream activation of eIF2α-CHOP and finally culminated into alleviation of autophagy response in cells (**Figures 2A–D**) and thereby prevented NPC-402 apoptosis as analyzed by Western blotting (**Figures 2E, F**) and immunostaining assay (**Figures 2I, J**). Cell viability analysis also supports that 4PBA retards NPC-402-induced cell death in B16F10 cells (**Figure 2G**), and cyclin D1 expression clearly explains the prevention of cell cycle arrest caused by exposure of B16F10 cells to NPC-402 (**Figure 2G**). Transcriptional factor CHOP also plays a highly significant role in ER stress-mediated apoptosis, as the sustained activation of UPR causes upregulation of CHOP *via* GRP78/ATF4 signaling pathway (42). Sustained CHOP upregulation increases the induction of autophagy that leads cells toward apoptosis. We silenced CHOP by using specific siRNA transfection in B16F10 cells and observed that knockdown of CHOP prevented the induction of autophagy that blocked cell death in NPC-402-treated, CHOP siRNA-transfected B16F10 cells compared to NPC-402 only-treated cells (**Figures 2K, L**).

ROS generation and ER stress along with UPR lead to oxidative and mitochondrial damage and induce cell death in cancer cells when treated with anticancer drugs (43, 44). Here, we observed that NPC-402 markedly increases ROS in B16F10 cells as analyzed by microscopy (**Figures 4A, B**) and flow cytometry assay (**Figure 4C**). It was found that NPC-402 reduced the Nrf-2 expression and modulated downstream redox signaling proteins SOD and catalase probably due to DNA damage and elevation of the oxidative stress response in B16F10 cells (**Figures 4D, E**). We also observed that antioxidant NAC diminished the effect of NPC-402 on oxidative stress (**Figures 4F–H**) and prevented NPC-402-induced cell death (**Figure 4I**).

Finally, we evaluated the effect of NPC-402 on cell death signaling. In melanoma therapy, target-specific cell death is very important for melanoma tumors (45). NPC-402 was found to be a potential cell death-inducing agent in melanoma cells through dysregulation of survival signaling pathways and activation of apoptotic signaling and prevented the proliferation and transformation of cancer cells into tumors. The proapoptotic effects of NPC-402 in B16F10 cells can be attributed to a change in ΔΨm (**Figures 3E, F**), PARP-1 cleavage, activation of caspases (**Figures 3G–J**), DNA damage (3D), and nuclear fragmentation (**Figure 3A**). We have also confirmed the release of cytochrome C from the mitochondrial inner membrane space that conveys that, upon treatment, NPC-402 induced a cascade of apoptotic events in B16F10 cells (**Figure 3C**). Taken together, these events constitute diverse mechanisms behind the anti-melanoma activity of NPC-402 in B16F10 cells through intrinsic and extrinsic apoptotic pathways.

Anticancer drugs are known to alter/hinder multiple intracellular signaling pathways in melanoma (46). Classically, MAPK signaling pathway plays a protective and pivotal role in response to varied external stimuli (47). Based on the findings of the present study, it was evident that NPC-402 decreased the activity of MAPK signaling axis in favor of increased apoptosis (**Figures S2D–S2G**). Furthermore, NPC-402 downregulated AKT survival signaling pathway (**Figures 4K, L**) albeit upregulated the expression of p-mTOR (**Figure 4L**), corroborating with growth inhibitory effects of NPC-402 in B16F10 melanoma cells. Moreover, NPC-402 is pharmacologically active through the i.p. route of drug administration. NPC-402 administration effectively inhibits B16F10 cell growth in the Matrigel plug assay. NPC-402 was equally effective as a standard clinical drug, dacarbazine, in retarding angiogenesis as was found through estimation of Hb through the Drabkin test (**Figures 5A–I**). These results suggest that NPC-402 inhibits VEGF-mediated angiogenesis, with relatively fewer toxic manifestations on normal cells.

## Conclusion

Overall, the present study supports the use of NPC-402 against melanoma cells as a potential therapeutic lead that may have relevance clinically. Furthermore, more mechanism-based and preclinical studies are required to confirm the therapeutic safety, efficacy, and potency of NPC-402 in its use as a promising melanoma drug.

## Author's note

We dedicate this work to the memory of Dr P.L. Sangwan, the Co-author of this article, who left for heavenly abode on 6th of August 2021. We will miss him for his uncanny knowledge of Natural Products Chemistry, for being a great human being, a great friend and a great collaborator.

## Data availability statement

The original contributions presented in the study are included in the article/**Supplementary Material**. Further inquiries can be directed to the corresponding author.

## Ethics statement

The animal study was reviewed and approved by Institutional Animal Ethics Committee, IAEC-IIIM, Jammu.

## Author contributions

LN, NS, US performed the experiments. KA, SR, SB and PS performed the synthesis of NPC402. ST made hypothesis, coordinated and procured the funding for the study. ST and LN wrote the MS.

## Acknowledgments

Senior Research Fellowship to LN by University Grants commission, New Delhi, India under NET-JRF scheme and Senior Research Fellowship to US by Department of science and technology vide no. IF-160982, New Delhi, India is acknowledged. This work was supported by council of scientific research and industrial research (CSIR), New Delhi, India vide project No GAP 2166. The manuscript has been passed through the research committee of the Institute CSIR-IIIM vide publication no: IIIM/IPR/00150.

## Conflict of interest

The authors declare that the research was conducted in the absence of any commercial or financial relationships that could be construed as a potential conflict of interest.

## Publisher’s note

All claims expressed in this article are solely those of the authors and do not necessarily represent those of their affiliated organizations, or those of the publisher, the editors and the reviewers. Any product that may be evaluated in this article, or claim that may be made by its manufacturer, is not guaranteed or endorsed by the publisher.

## Abbreviations

NPC-402, 3-O-prenyl glycyrrhetinic acid;MAP kinase, mitogen-activated protein kinase; AKT, protein kinase B; JNK, c-Jun N-terminal kinase; ER, endoplasmic reticulum; UPR, unfolded protein response; 4PBA, 4-phenylbutyric Acid; siRNA, small interfering RNA; CHOP/GAD153, C/EBP homologous protein; NAC, N-acetylcysteine; GRP78/Bip, glucose-regulated protein 78; IRE1α, inositol-requiring transmembrane kinase/endoribonuclease 1α; PERK, protein kinase R-like endoplasmic reticulum kinase; ATF6, activating transcription factor 6; Bcl-2, B-cell lymphoma 2; Bim, Bcl-2 homology 3-only protein; Bax, BCL2-associated X; ATF4, activating transcription factor 4; MEK, mitogen-activated protein kinase kinase; ERK1/2, extracellular signal-regulated kinase 1/2; Nrf-2, nuclear factor erythroid 2-related factor 2; Cu/Zn SOD, copper/zinc superoxide dismutase; P62, ubiquitin-binding protein**;** GAPDH, glyceraldehyde 3-phosphate dehydrogenase
