# Supplementary material for: Synthesis and anti-melanoma effect of 3-O-prenyl glycyrrhetinic acid against B16F10 cells via induction of endoplasmic reticulum stress-mediated autophagy through ERK/AKT signaling pathway
Source: Front Oncol. 2022 Aug 2;12:890299. doi: 10.3389/fonc.2022.890299 (PMC9380594; doi:10.3389/fonc.2022.890299)
Supplement: Supplementary file 1 [file DataSheet_1.pdf]

(S-1A)

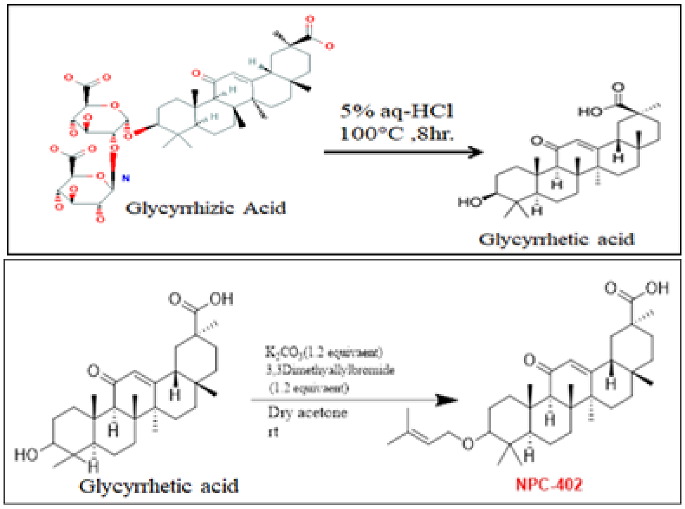

(S-1B)

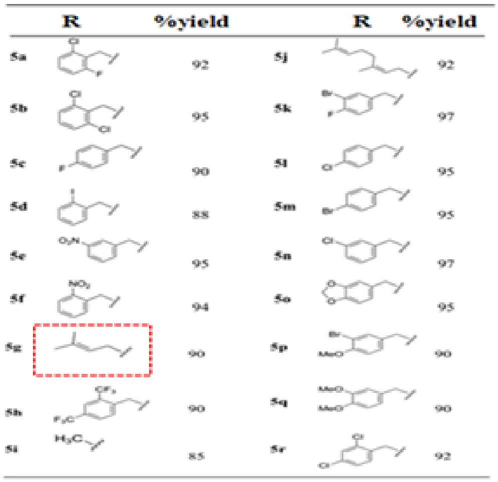

(S-1C)

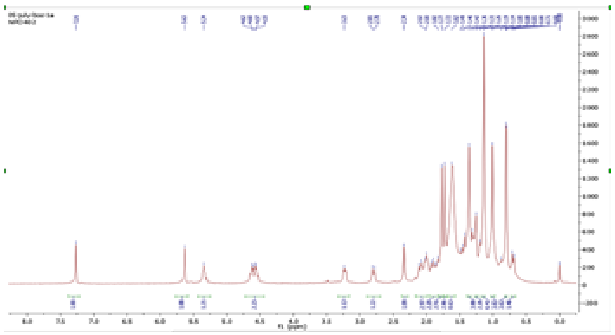

(S-1D)

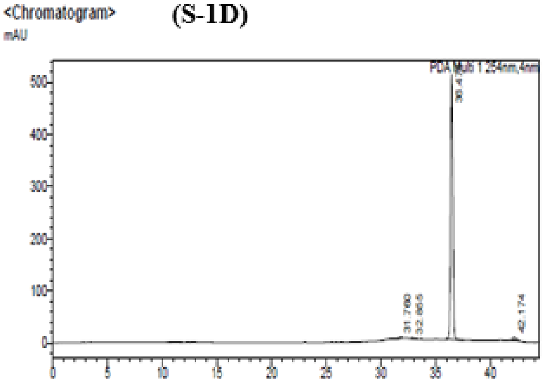

Supplementary: Figure 1

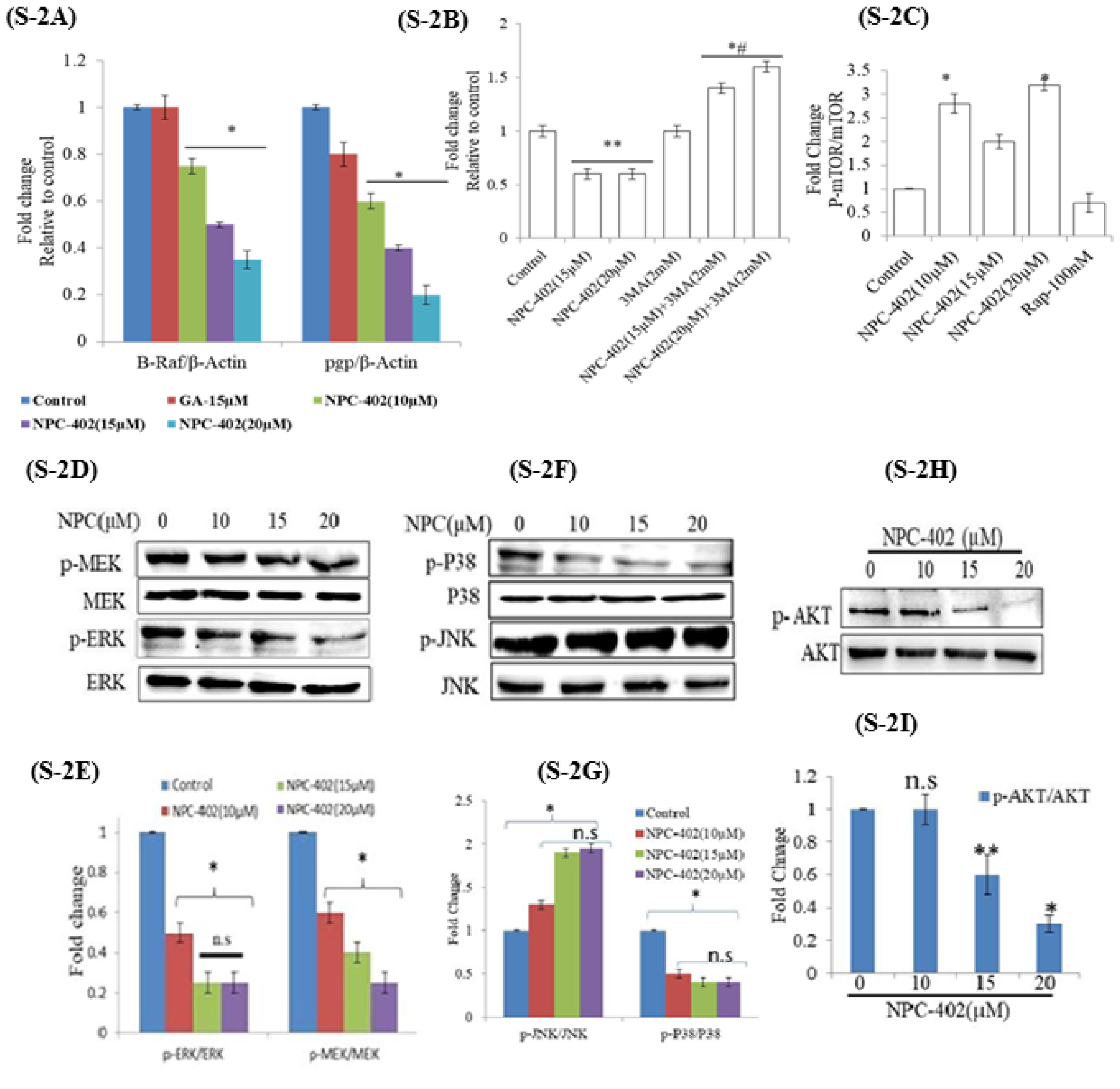

Supplementary: Figure 2

S-3

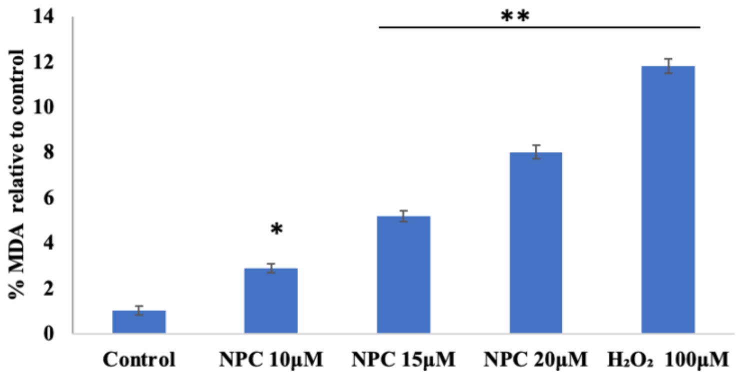

Supplementary: Figure 3

Supplementary Figure 1: Synthesis, analysis and purity profile of *3-(3-Methyl-but-2-enyloxy)-11-oxo-Olean-12-ene-29-oic acid* (NPC-402).

(S-1A) represents the synthetic scheme for different analogs *O*-alkylated/benzylated Glycyrrhetenic acid. **(S-1B** Different Analogs of *O*-alkylated/benzylated Glycyrrhetenic acid with yield percentage. **(S-1C)** Represents the NMR Spectrum of Proton ($H^{1)}$ of NPC-402. **(S-1D)** Represents the HPLC- chromatogram of NPC-402.

Supplementary Figure 2:

(**S-2A, 2B ,2C**) Bar graph represents densitometry evaluation of proteins shown in figure 4J,4K and 4L using Bio-Rad Image Lab™ Software in B16F10 cells treated with NPC-402 (*represents *p<0.001*; ** *p<0.01,* control vs NCP402 treated). **(S-2D)** Immuno-Blots represent the Protein Expression of p-MEK/MEK, p-ERK/ERK using β-actin as loader control in B16F10 cells treated with NPC-402. **(S-2E)** Bar graph represents densitometry evaluation of Protein Expression of p-MEK/MEK and p-ERK /ERK using Bio-Rad Image Lab™ Software in B16F10 cells treated with NPC-402. **(S-2F)** Immuno-Blot represents the Protein Expression of p-P38, P38, p-JNK and JNK using β-actin as loader control in B16F10 cells treated with NPC-402. **(S-2G)** Bar graph represents densitometry evaluation of western blots p-JNK/JNK and p-P38/P38 using Bio-Rad Image Lab™ Software in B16F10 cells treated with NPC-402. **(S-2H)** Immuno-Blot represents the Protein Expression of p-AKT and AKT using β-actin as loader control in B16F10 cells treated with NPC-402. **(S-2I)** Bar graph represents densitometry evaluation of p-AKT/AKT using Bio-Rad Image Lab™ Software in B16F10 cells treated with NPC-402

Supplementary Figure 3:

Figure S-3 represents the MDA levels in B16F10 cells after 24 hours of treatment with **NPC-402** quantified by Lipid peroxidation assay. (*represents $p<0.001$; ** $p<0.01$, control vs NCP-402 treated).
