# Supplementary figures and images for: Synthesis and anti-melanoma effect of 3-O-prenyl glycyrrhetinic acid against B16F10 cells via induction of endoplasmic reticulum stress-mediated autophagy through ERK/AKT signaling pathway
Source: Front Oncol. 2022 Aug 2;12:890299. doi: 10.3389/fonc.2022.890299 (PMC9380594; doi:10.3389/fonc.2022.890299)

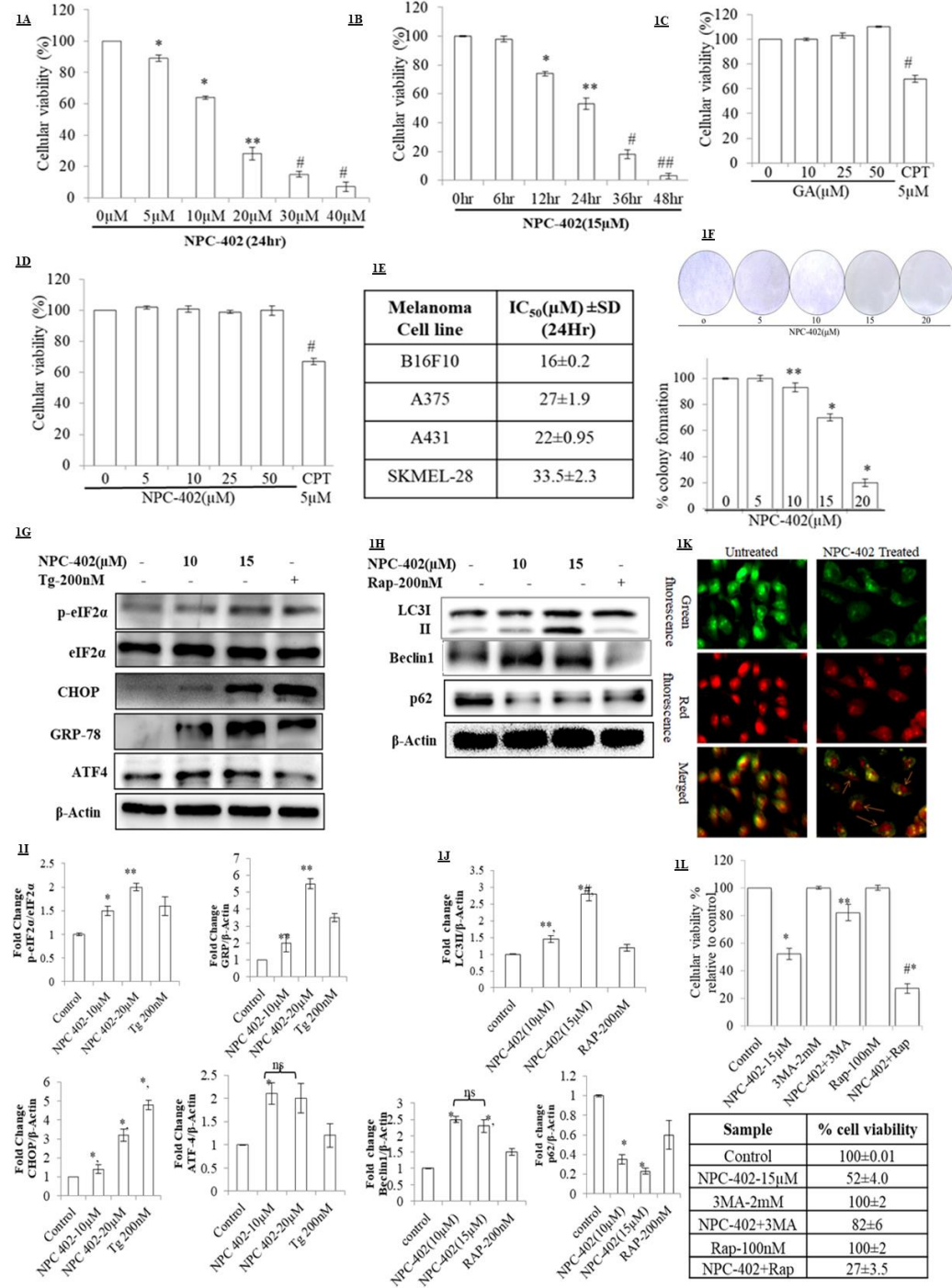

Figure 1

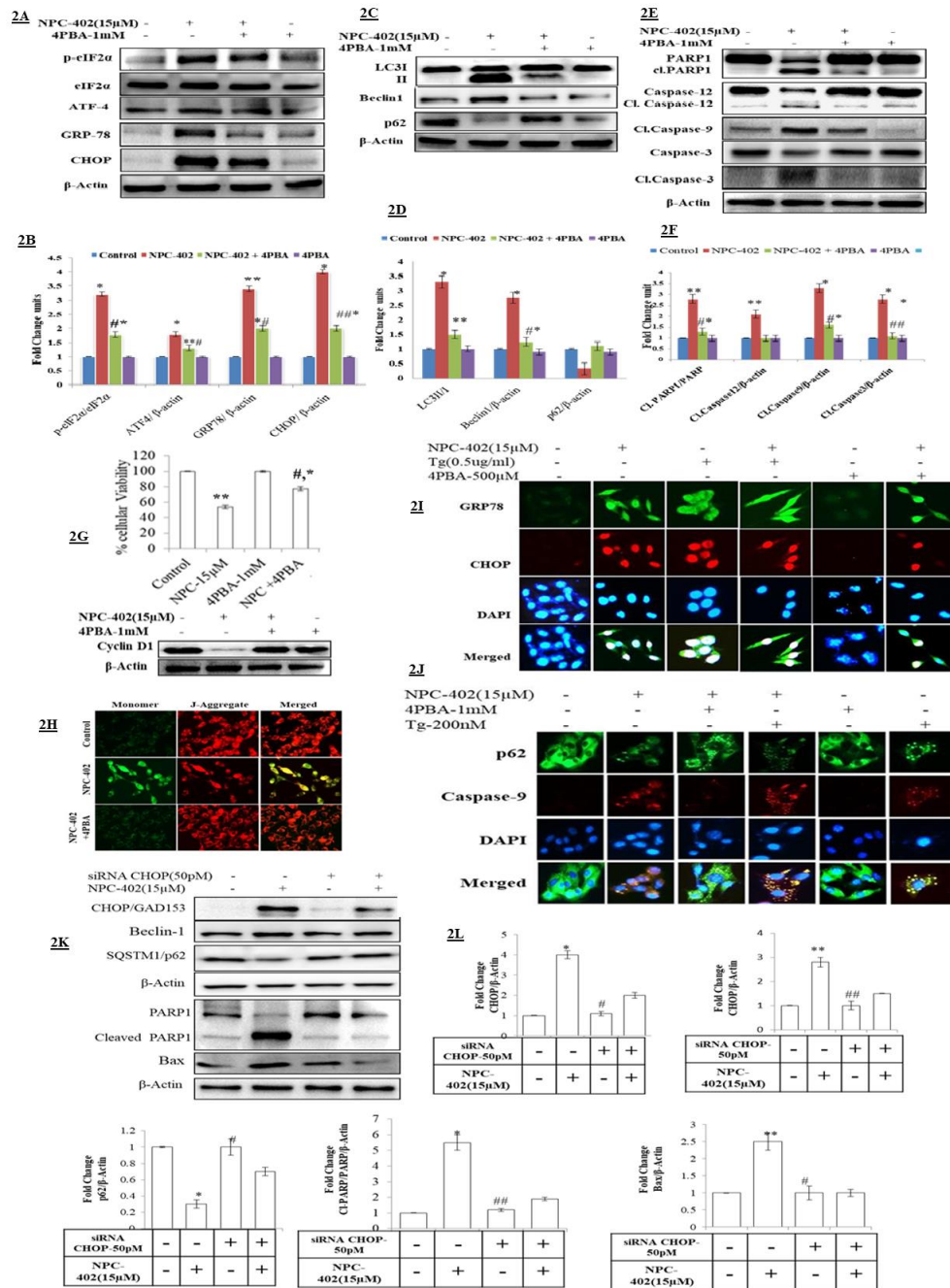

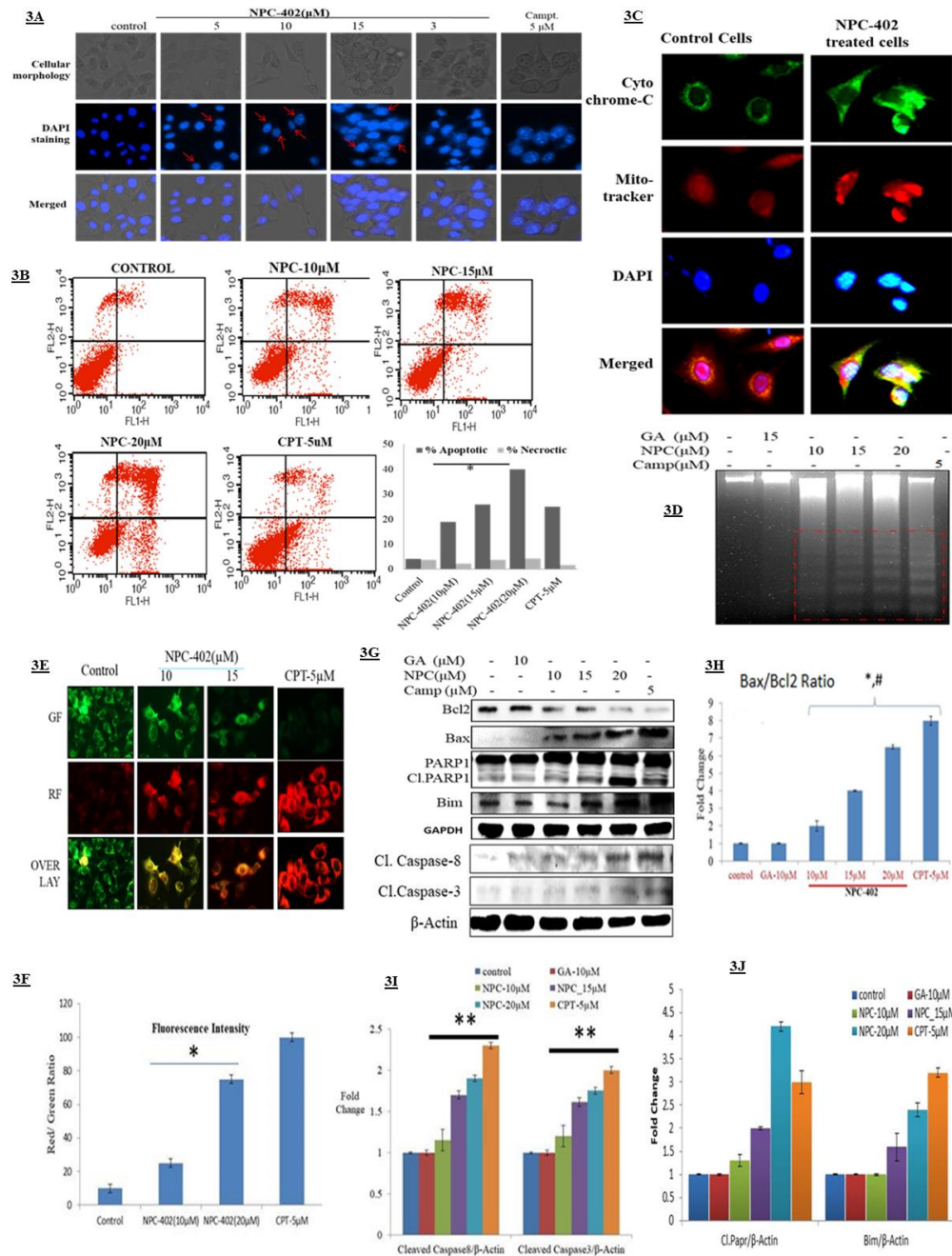

**Figure: 3**

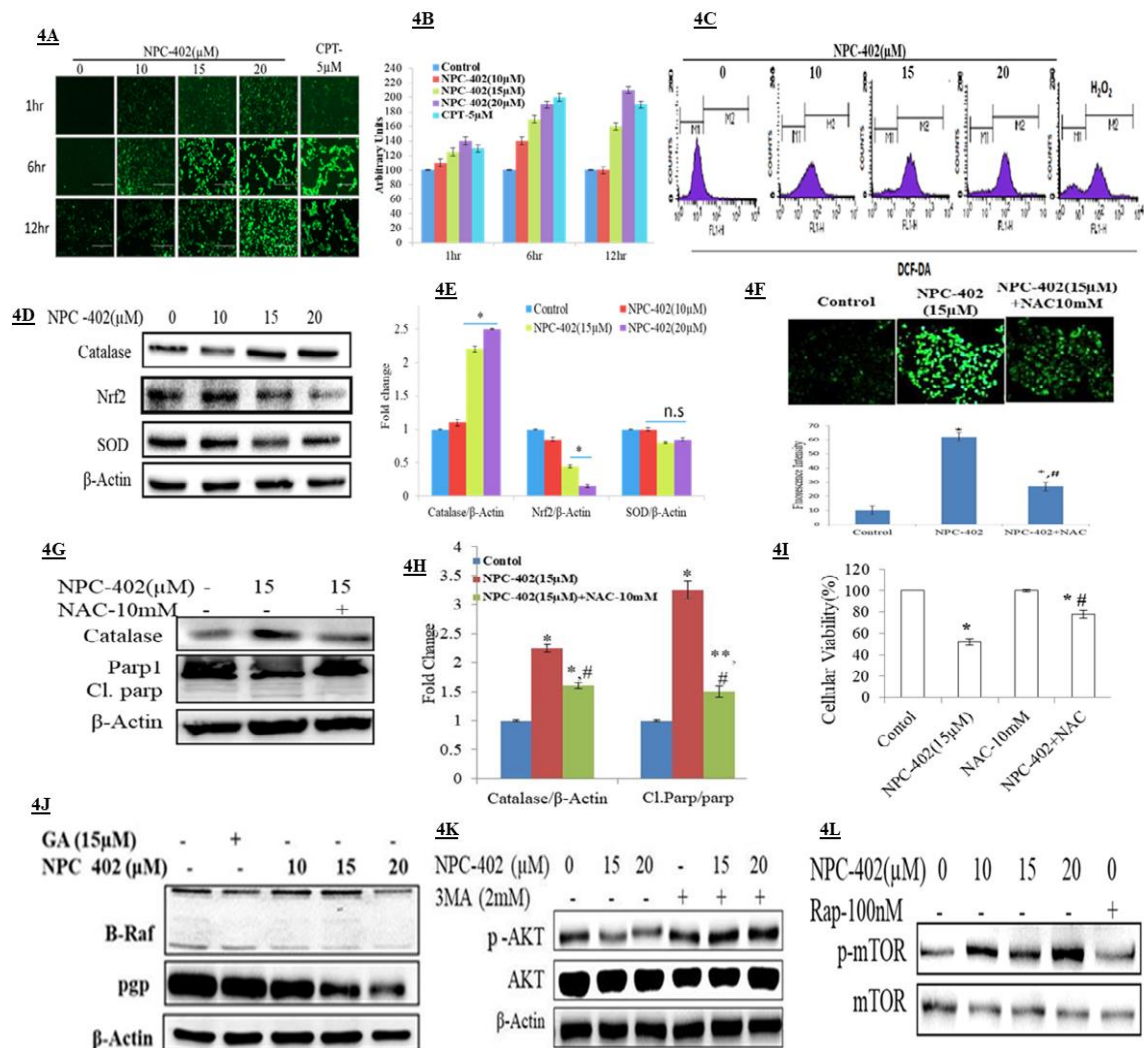

**Figure : 4**

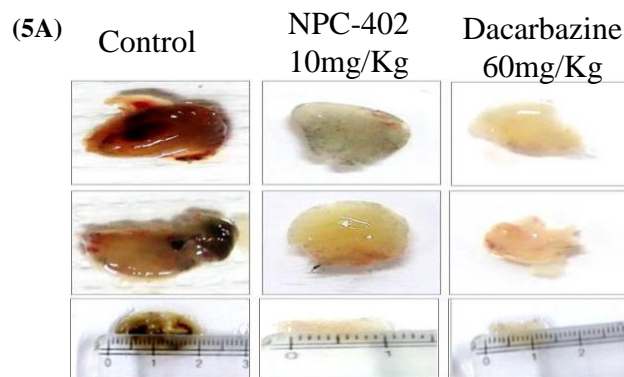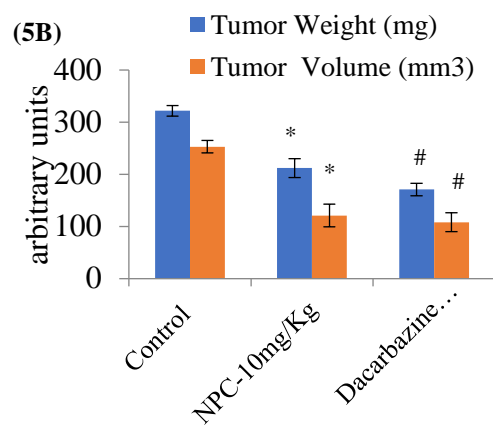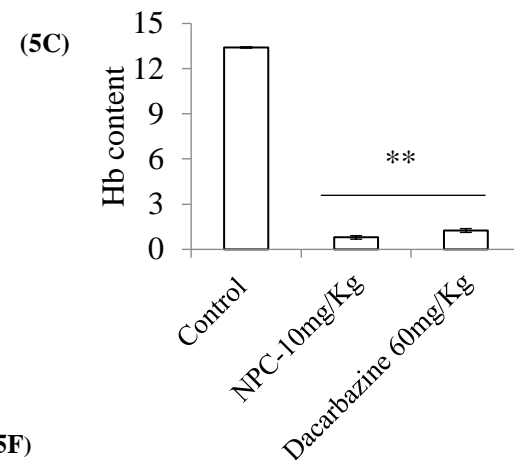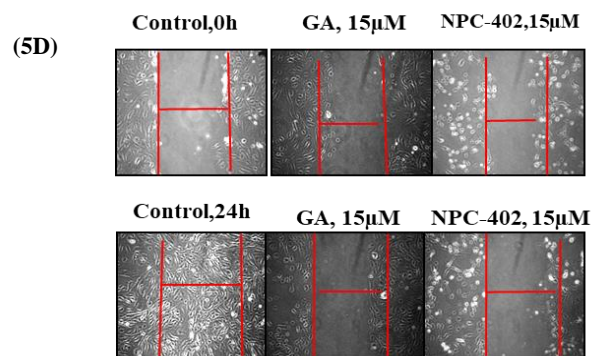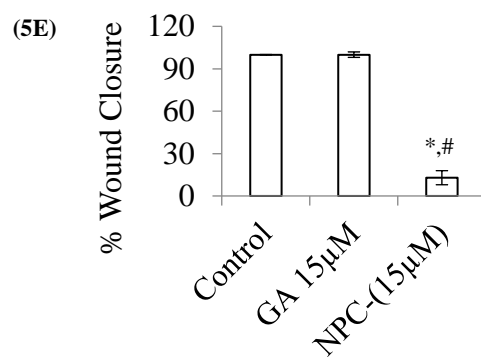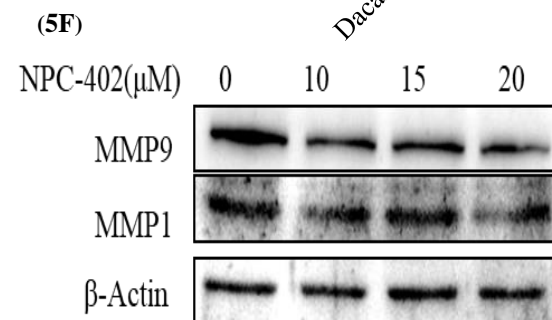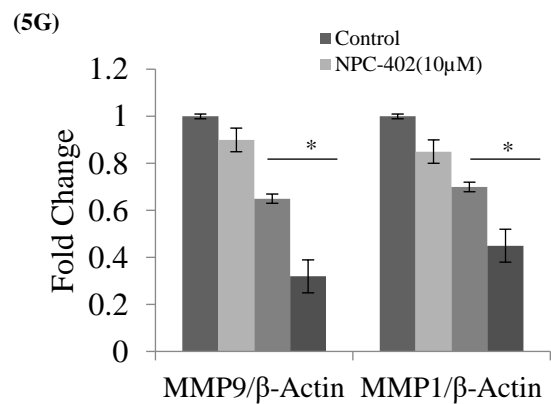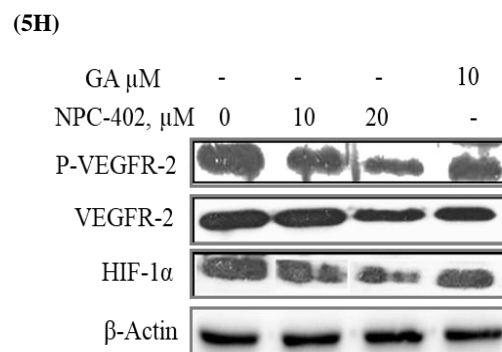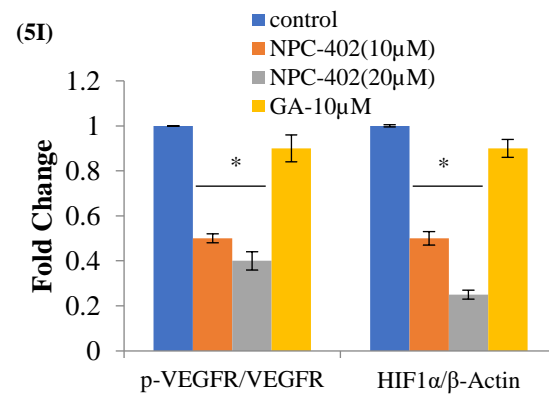

Supplement: Supplementary file 2 [file DataSheet_2.pdf]
